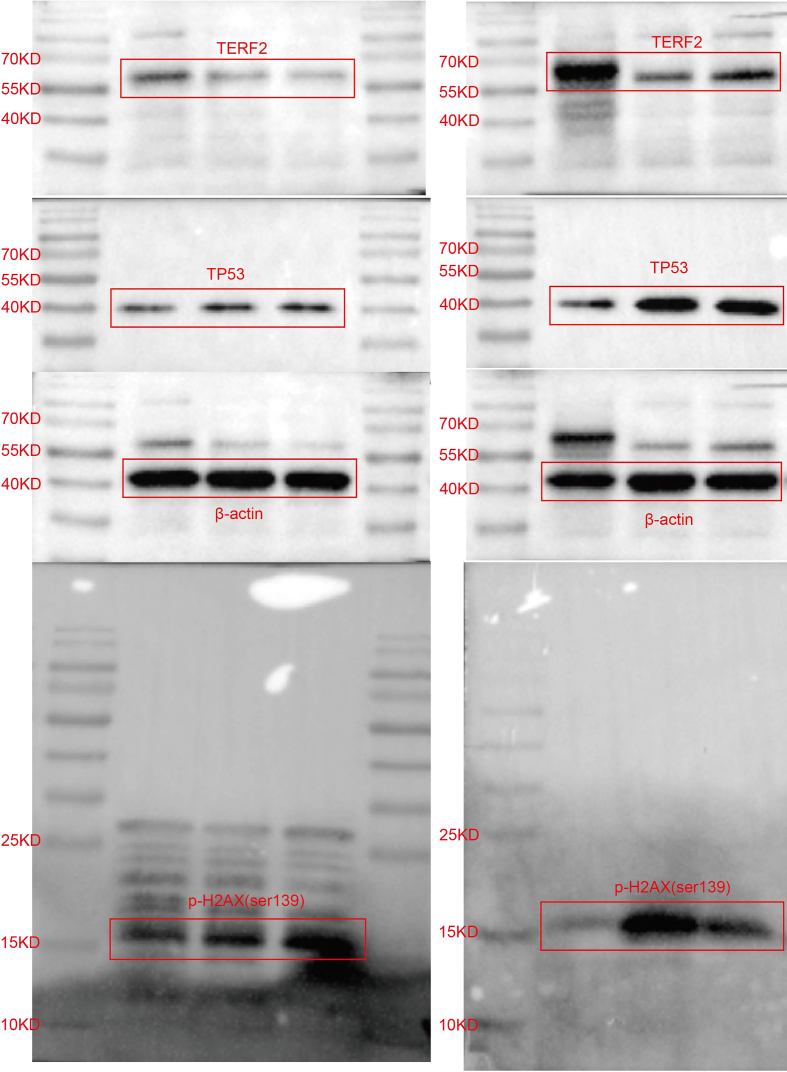# Expression and functional analyses of TERF2 in esophageal carcinoma

**DOI:** 10.1016/j.heliyon.2024.e38040

**Published:** 2024-09-18

**Authors:** Lihua Yao, Xinlu Wang, Zihao Wang, Xiaozhong Wang, Xiaolan Guo

**Affiliations:** a Department of Clinical Laboratory, Affiliated Hospital of North Sichuan Medical College, Nanchong, 637000, PR China; b Department of Clinical Laboratory, The Second Affiliated Hospital of Nanchang University, Nanchang, 330006, PR China

**Keywords:** Esophageal cancer, TERF2, Biomarker, Bioinformatics analysis

## Abstract

**Background:**

Esophageal cancer (ESCA) is a prevalent malignancy with a high incidence of morbidity and mortality, particularly in Asia. Telomeric Repeat-binding Factor 2 (TERF2) is a crucial component of the telomere-binding protein complex that maintains telomere stability. Aberrant TERF2 expression has been implicated in tumorigenesis, however, its specific role in ESCA remains largely unexplored.

**Methods:**

The expression levels of TERF2 were assessed in esophageal squamous cell carcinoma (ESCC) samples using RT-PCR, IHC, and Western blotting **(**WB). Serum tumor marker concentrations were determined via electrochemiluminescence immunoassay (ECLIA) and chemiluminescent microparticle immunoassay (CMIA). Bioinformatics analyses were employed to elucidate TERF2's function in EC. The impact of TERF2 on ESCC cell proliferation was evaluated through cell counting kit-8 (CCK8) assays and flow cytometry.

**Results:**

TERF2 protein and mRNA expression were elevated in ESCC tissues and correlated with age, sex, cancer stage, tumor grade, lymph node metastasis (LNM), and tumor histology. Univariate Cox regression analysis indicated TERF2 was an independent prognostic factor for overall survival (OS). TERF2 mRNA levels were associated with serum levels of carcinoembryonic antigen (CEA), cytokeratin 19 fragment (CYFRA21-1), and tissue polypeptide antigen (TPA) in patients with ESCC. Immune infiltration and chemokine profiles were linked to TERF2 expression in ESCA. TERF2 is involved in regulating ESCC cell proliferation may through the DDR/P53 signaling way.

**Conclusions:**

TERF2 is overexpressed in ESCA and contributes to ESCC cell proliferation may via DDR/TP53 signaling pathway. These results suggest that TERF2 may serve as a potential target for developing treatments and diagnostic biomarker for ESCA.

## 1 Introduction

Esophageal cancer ranks as the seventh most common cancer in males and the sixteenth in females, with an estimated 511,054 new cases reported globally in 2022. Approximately 80 % of these cases occur in Asia or Africa. ESCA is categorized into two primary subtypes: esophageal squamous cell carcinoma (ESCC), predominantly developing in the upper esophagus, and esophageal adenocarcinoma (EAC), typically arising at the gastroesophageal junction. Globally, ESCC is a frequent histological type, particularly prevalent in East Asian countries such as China and Japan [1]. Despite advancements in perioperative care, surgical techniques, and multimodal treatment approaches [2], ESCA continues to exhibit a high mortality rate. While early-stage ESCA often carries a favorable prognosis, advanced disease is associated with poor outcomes and low survival rates [3]. To enhance early detection and treatment, the identification of novel molecular targets driving ESCA initiation and progression is essential.

Telomeres, repetitive DNA sequences situated at the termini of linear chromosomes, are crucial for preserving chromosomal integrity [4]. They are protected by a six-protein complex known as shelterin, comprising TERF1, TERF2, RAP1, TPP1, POT1, and TIN2. These shelterin components possess distinct functions, primarily inhibiting DNA damage repair pathways to safeguard telomere stability [5]. A substantial body of research has demonstrated that shelterin proteins can undergo upregulation or downregulation at both the protein and transcriptional levels across various cancer types. As a pivotal component of shelterin, TERF2 is frequently overexpressed in multiple cancers, underscoring its critical role in tumorigenesis and progression [[6], [7], [8], [9], [10], [11], [12], [13], [14]]. Nonetheless, the expression, function, and diagnostic utility of TERF2 in esophageal cancer remain relatively understudied.

This study aimed to quantify TERF2 expression levels, evaluate their diagnostic utility, explore their biological role, and examine the influence of TERF2 on cell proliferation in esophageal cancer. Overall, our results might provide evidence of TERF2 as a potential diagnostic biomarker and therapeutic target for ESCA patients.

## 2 Materials and methods

### 2.1 Patient population and ethical approval

This study gained approval from the Medical Ethics Committee of the Affiliated Hospital of North Sichuan Medical College (File No. 2022ER461-1). Written informed consent was obtained from all participants prior to their involvement. Samples of ESCC and corresponding non-cancerous tissues were collected at the Affiliated Hospital of North Sichuan Medical College.

### 2.2 Expression analysis

To evaluate TERF2 mRNA expression across various cancer types, the Tumor Immune Estimation Resource (TIMER) (http://timer.cistrome.org/) and TCGA database (https://cancergenome.nih.gov/) were employed. Subsequently, the UALCAN (http://ualcan.path.uab.edu/index.html) was utilized to investigate the correlation between TERF2 expression levels and clinicopathological features, encompassing tumor stage, sex, age, race, and tumor histology.

### 2.3 Survival curve analysis

The R package “survival” was employed to conduct univariate and multivariate Cox regression analyses to evaluate the independent prognostic value of TERF2 in relation to additional clinical variables, including sex, stage, and TNM staging. Receiver operating characteristic (ROC) curves were generated using R, and the optimal cut-off value for TERF2 was determined by identifying the Youden index.

### 2.4 Western-blotting and qRT-PCR assays

qRT-PCR and WB were performed to quantify TERF2 mRNA and protein expression in tissues and cells, respectively, as previously detailed [15]. The following primers were used to amplify TERF2 mRNA: 5′-GACCTTCCAGCAGAAGATGCT-3’ (forward) and 5′-GTTGGAGGATTCCGTAGCTA-3’ (reverse). β-actin served as a reference gene with the primer: 5′-GGACTTCGAGCAAGAGATGG-3’ (forward) and 5′-AGCACTGTGTTGGCGTACAG-3’ (reverse). Primary antibodies against TERF2 (1:500), TP53 (1:500), H2A.X (1:500), and GAPDH (1:5000) were procured from Cell Signaling Technology (CST; Danvers, MA, USA).

### 2.5 Immunohistochemistry (IHC)

As previously described [16], IHC for TERF2 was performed an anti-TERF2 (1:50) antibody (Abcam, Cambridge, UK). An overall IHC score was measured by multiplying the intensity and extent scores, ranging from 0 to 12. Staining was considered negative for scores 0–4 and positive for scores 5–12. Two independent pathologists blinded to patient grouping assessed staining intensity and extent.

### 2.6 Quantification of tumor markers

Serum concentrations of CEA, SCC, CYFRA21-1, and TPA were quantified using reagent kits on an electrochemiluminescence immunoassay platform (Roche 620, Basel, Switzerland). Furthermore, serum SCC levels were specifically determined using an SCC reagent kit on the ARCHITECT i2000 system (Abbott Laboratories, USA) via a chemiluminescent microparticle immunoassay.

### 2.7 Immune infiltration analysis

The TIMER2.0 was utilized to explore the correlation between TERF2 level in ESCA and tumor-infiltrating immune cells (TIICs). Additionally, the Tumor Immune System Interactions (TISIDB) platform (http://cis.hku.hk/TISIDB/index.php) was utilized to analyze the correlation between TERF2 and ESCA-associated chemokines.

### 2.8 Gene network establishment

GeneMANIA (http://www.genemania.org) leverages existing genomic and proteomic data to generate functional gene hypotheses [17]. To investigate the functional relationships of the TERF2, we employed the GeneMANIA Manage app to import it into Cytoscape software (version 3.8.2) [18]. This facilitated the visualization of a functional interaction network of TERF2 with relevant genes.

### 2.9 Functional annotation as well as pathway enrichment

In this study, the Database for Annotation, Visualization and Integrated Discovery (DAVID, https://david.ncifcrf.gov/) was employed for integrative gene functional enrichment and biological pathway analysis [19], and the results were visualized as histogram charts.

### 2.10 Cell culture and transfection with small interfering RNA (siRNA)

ESCC cell lines (Eca109 and Kyse150) were obtained from the Cell Bank of the Shanghai Institute of Cell Biology (Shanghai, China), and cultured in RPMI-1640 medium (Gibco, Grand Island, NY, USA) supplemented with 10 % fetal bovine serum (FBS; Gibco) at 37 °C. Lipofectamine 2000 (Invitrogen, Waltham, MA, USA) was employed to deliver TERF2 siRNA (GenePharm, Shanghai, China) targeting the following sequences: 5′-CCACUGGAAUCAGCUAUCATT-3′ (F), 5′- UGAUAGCUGAUUCCAGUGGTT-3′ (R), 5′-GAAGACAGUACAACCAAUATT-3′ (F), and 5′-UAUUGGUUGUACUGUCUUCTT-3′ (R).

### 2.11 Cell proliferation assay

Cell viability was measured using the CCK-8 assay, as previously detailed [20]. All measurements were performed in triplicate.

### 2.12 Cell cycle assay

The cells were fixed in 70 % ethanol for 2 h. Subsequently, the cells were centrifugated and resuspended in propidium iodide (PI) solution for analysis by flow cytometry.

### 2.13 Statistical analysis

Non-parametric Wilcoxon tests were employed to compare mRNA levels between tumor and non-carcinoma specimens. Fisher's exact test was utilized to assess the association between TERF2 expression and clinicopathological characteristics. Spearman's correlation analysis was conducted to evaluate the correlation between TERF2 mRNA expression and serum tumor marker levels. Statistical analyses were performed using R software and GraphPad Prism 9.0 (GraphPad, San Diego, CA, USA). Statistical significance was defined as a *p*-value less than 0.05.

## 3 Results

### 3.1 TERF2 transcriptional and protein levels among ESCA patients

To investigate TERF2 expression patterns in patients with ESCA, the present study analyzed 33 tumor types and corresponding non-carcinoma samples using the TIMER database. ESCA samples exhibited significantly increased TERF2 mRNA levels compared to non-carcinoma controls, a finding independently validated by TCGA database analysis (Fig. 1A and B). To further corroborate these observations, RT-qPCR was performed on 65 ESCC tumors and adjacent non-tumor tissues, confirming elevated TERF2 mRNA expression in tumor samples (Fig. 1C). Western blot analysis of 39 paired ESCC samples demonstrated 27 tumor tissues significantly higher TERF2 protein levels than non-tumor controls (Fig. 1D and E, Supplementary Fig. 1). IHC analysis revealed pronounced TERF2 overexpression in cancer tissues (Fig. 1F and G). Collectively, these results consistently demonstrate upregulated TERF2 expression at both the mRNA and protein levels in ESCA.

**Fig. 1:**
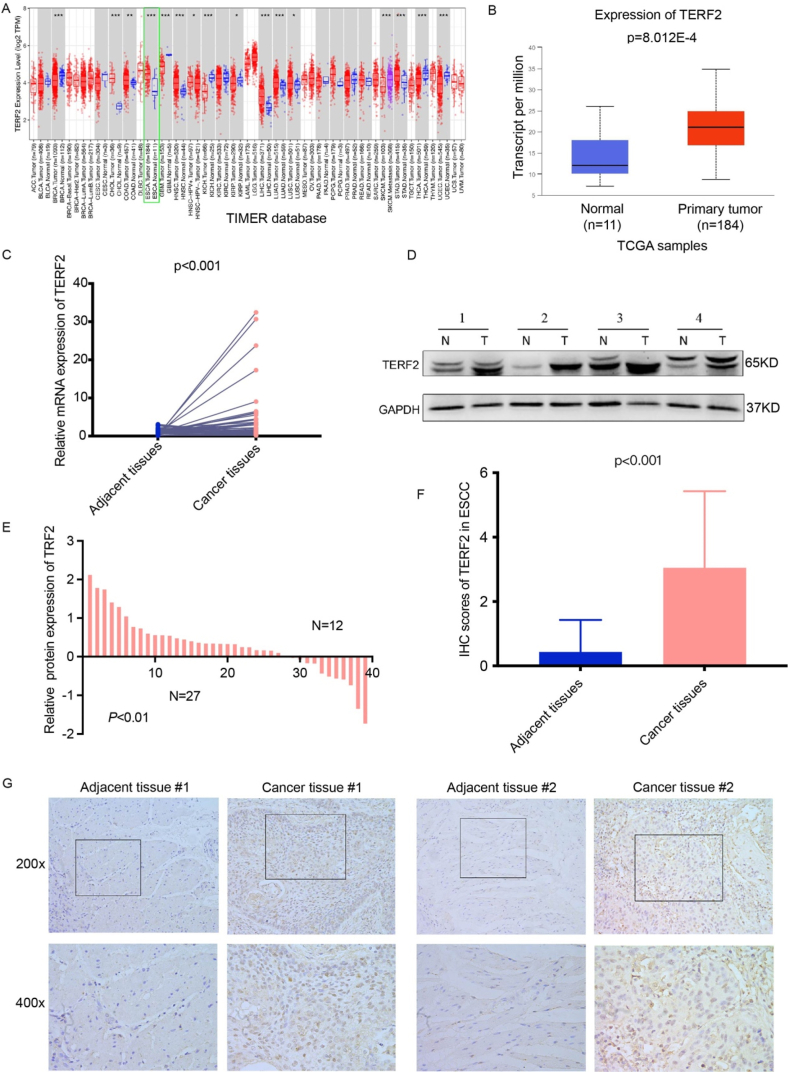
**Transcriptional and protein expression studies of TERF2 among ESCA patients.** A. TERF2 transcriptional levels within 33 TIMER-derived cancer types. B. TERF2 mRNA levels in TCGA-ESCA tissues compared with normal samples. Red and blue stand for up-regulated and down-regulated levels. C. TERF2 mRNA level analyzed by qRT-PCR in 65 ESCC patients. D-F. The TERF2 protein levels examined through WB within 39 ESCC patients. G. The protein level of TERF2 determined by IHC in 44 ESCC patients. (For interpretation of the references to color in this figure legend, the reader is referred to the Web version of this article.)

### 3.2 Correlation of TERF2 transcriptional levels with clinicopathological characteristics of ESCA patients

**Fig. 2:**
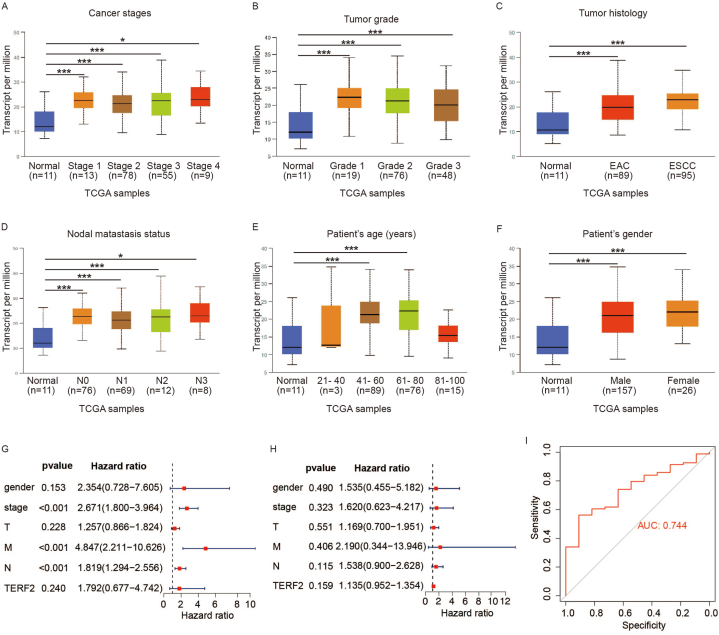
**Correlation of TERF2 transcriptional levels with clinicopathological characteristics of ESCA patients.** A-F. TERF2 transcriptional expression related to age, sex, tumor grade, cancer stage, tumor histology, and LNM. ∗*p* < 0.05, ∗∗*p* < 0.01, ∗∗∗*p* < 0.001. G-H. Univariate and multivariate regression analyses performed on TERF2. I. The prognostic significance of TERF2 examined using ROC curve analysis.

The relationships between clinical factors and TERF2 transcriptional levels in patients with ESCC were analyzed using UALCAN. Upregulated TERF2 expression was significantly associated with cancer stages 1–4 (Fig. 2A), tumor grades 1–3 (Fig. 2B), and lymph node metastasis statuses N0, N2, N3, and N4 (Fig. 2D). Additionally, increased TERF2 mRNA levels correlated with tumor histology, age, and sex compared to normal tissues (Fig. 2C–F). Notably, while no significant difference in TERF2 expression was observed between cancer and normal tissues in patients aged 21–40 years, a marked disparity emerged in the 41–100 age group. This discrepancy may be attributed to the later onset of esophageal cancer and the limited sample size (n = 3) in the younger cohort. To evaluate TERF2's prognostic value, univariate and multivariate regression analyses were performed using the TCGA cohort. Univariate Cox regression analysis revealed TERF2 as an independent predictor of OS in ESCC patients (Fig. 2G and H). TERF2 achieved an area under the ROC curve (AUC) value of 0.744 (Fig. 2I), suggesting its potential as a diagnostic biomarker for ESCA. These findings shown a significant correlation between TERF2 levels and clinicopathological characteristics in ESCC patients.

### 3.3 Correlations between TERF2 mRNA level and clinical features and tumor markers in ESCC patients

Next, we investigated the correlation between TERF2 mRNA levels and the clinical characteristics of 65 ESCC patients. As shown in Table 1, TERF2 upregulation was significantly associated with TNM stage (*p* = 0.016). No significant correlations were observed with sex, differentiation, or lymph node metastasis. This outcome exhibits a certain degree of discrepancy with the findings derived from the TCGA database, which may potentially be attributed to the relatively small sample size in our study.

**Table 1:** Association of clinicopathological data and expression of TERF2 in ESCC patients.

Patient characteristics	TERF2 expression		X^2^	p-value
	High (n = 43)	Low (n = 22)		
Age			0.910	0.514
<65	22	8		
≥65	21	14		
Gender			0.064	1.000
Male	32	17		
Female	11	5		
Tumor size			2.456	0.236
<3 cm	10	7		
3–5 cm	29	15		
>5 cm	4	0		
Tumor location			2.258	0.745
Upper	5	4		
Middle	33	13		
Lower	5	5		
TNM stage			6.687	0.016∗
I–II	30	8		
III–IV	13	14		
Lymph node metastasis			0.005	1.000
Yes	18	9		
N	25	13		
Differention			2.476	0.256
Well	13	8		
Moderate	19	12		
Poor	11	2		

Using a Fisher's exact test. The p-value was set at 0.05 and ∗ indicates p < 0.05.

**Fig. 3:**
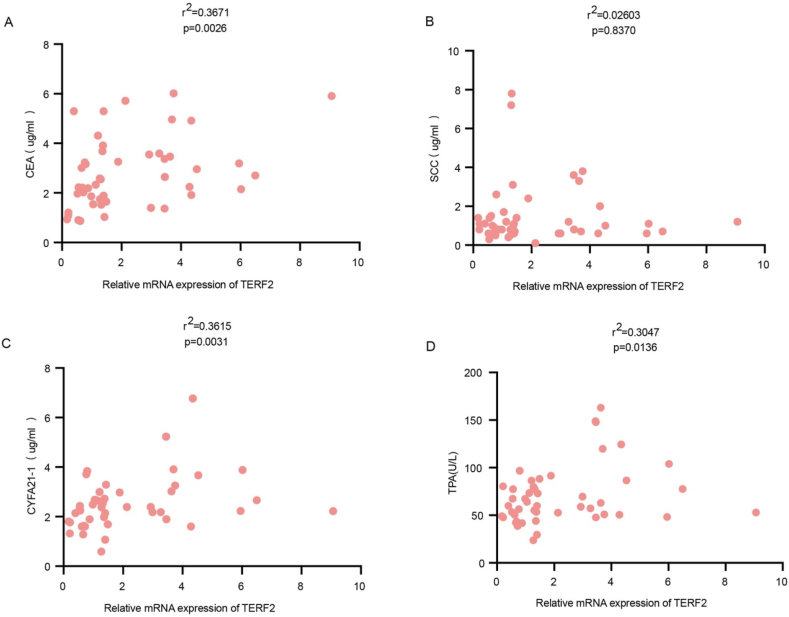
**Correlation of mRNA expression of TERF2 and tumor markers in ESCC patients.** A-D. The mRNA expression of TERF2 correlated with the serum level of CEA, SCC, CYFRA21-1, and TPA.

Furthermore, we examined the relationship between TERF2 mRNA levels and serum tumor markers. As depicted in Fig. 3, positive correlations were observed between TERF2 mRNA levels and serum levels of CEA, CYFRA21-1, and TPA (Fig. 3A and 3C-D). In contrast, no significant association was found with SCC levels (Fig. 3B). These results suggest that TERF2 may be a potential biomarker complementary to conventional tumor markers in evaluating ESCC.

### 3.4 Immune infiltration and chemokines related to TERF2 levels among ESCA patients

**Fig. 4:**
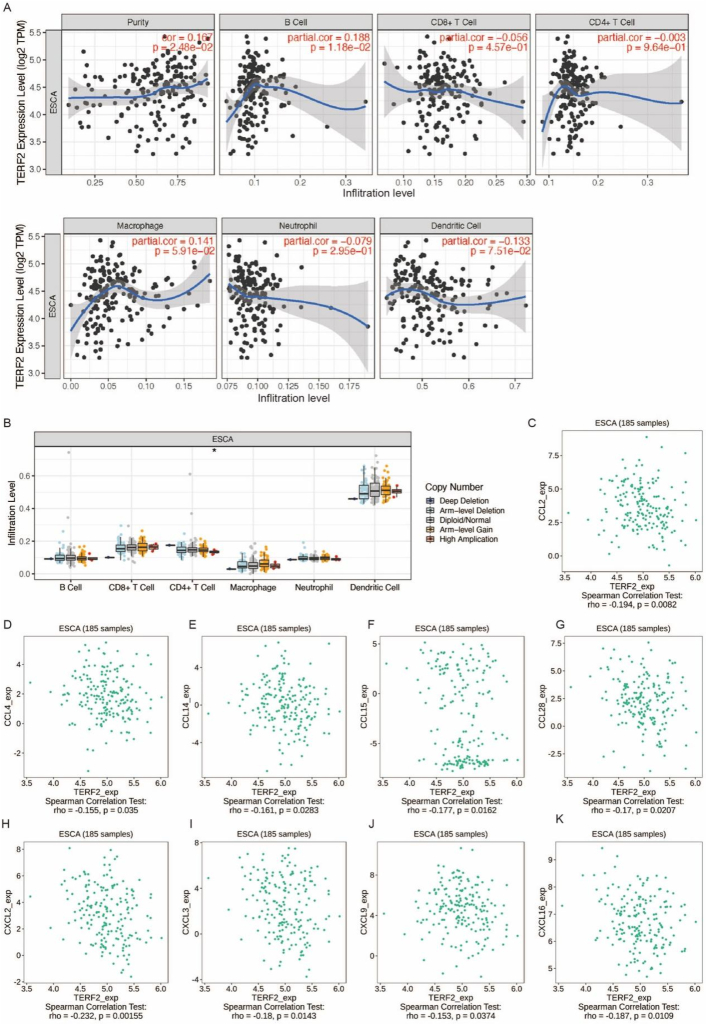
**Immune infiltration and chemokines related to TERF2 levels among ESCA patients.** A. Expression of TERF2 correlated with infiltrating immune cells examined based on the TIMER database. B. TERF2 copy number associated with immune infiltration cells analyzed by TIMER database. C-K. Association of TERF2 level with chemokines analyzed based on TISIDB database. ∗*p* < 0.05.

The relationship between TERF2 expression and immune infiltration was investigated using the TIMER database. Fig. 4A demonstrates a significant correlation between TERF2 levels and B cell infiltration (*p* = 0.0118), as well as tumor purity (*p* = 0.0248). Additionally, a significant correlation was observed between TERF2 copy number and CD4^+^ T cell infiltration in ESCA (Fig. 4B). To further explore the relationship between TERF2 and TIICs, the GEPIA database was employed to assess the correlation between TERF2 expression and immune marker levels. Table 2 summarizes the analysis of a broad spectrum of TIICs, including B cells, CD8^+^ T cells, general T cells, exhausted T cells, natural killer (NK) cells, neutrophils, mast cells, and T helper cell subsets (Th1, Th2, Th17, and Tfh). Significant correlations were identified between TERF2 levels and Th2 (TNF-α), Th17 (STAT3), and Tfh (BCL6) markers in ESCC tumor samples. In non-carcinoma samples, associations were observed between TERF2 levels and NK cell (KIR2DL1, KIR3DL1), Th2 (STAT5A, STAT6), neutrophil (CD11b), Th17 (STAT3), Tfh (BCL6), mast cell (CPA3, TPSAB1), and T-cell exhaustion (TIM-3) markers. Furthermore, ESCA tumor samples exhibited marked associations between TERF2 levels and a comprehensive panel of immune markerssuch as CD2, CD3E, CD8A, CD8B, CD19, KIR2DL4, KIR3DL2, KIR3DS4, CD11b, CCR7, T-bet, STAT3, STAT4, STAT5A, STAT6, BCL6, PD-1, LAG3, CTLA4, TPSB2, TIM-3, CPA3, MS4A2, and HDC. These findings collectively suggest the impact of TERF2 on the immune microenvironment (TME) in ESCA.

**Table 2:** The association of TERF2 expression and immune markers in GEPIA.

Description	Gene markers			ESCA			
		Tumor		Normal		GETX	
		R	P	R	P	R	P
CD8+T cell	CD8A	−0.086	0.25	0.13	0.67	0.19	0.0017
	CD8B	−0.14	0.063	−0.011	0.97	0.18	0.0023
T cell (general)	CD2	−0.14	0.069	0.075	0.81	0.22	0.00031
	CD3E	−0.13	0.082	0.086	0.78	0.21	0.00045
B cell	CD19	−0.044	0.56	0.024	0.94	0.13	0.034
	CD79A	−0.12	0.11	−0.23	0.44	0.042	0.49
Natural killer cell	KIR2DL1	−0.059	0.43	0.6	0.031	0.095	0.12
	KIR2DL3	−0.054	0.47	0.35	0.25	0.086	0.16
	KIR2DL4	0.01	0.89	−0.063	0.84	0.18	0.0024
	KIR3DL1	−0.043	0.57	0.66	0.014	0.11	0.065
	KIR3DL2	−0.0044	0.95	0.0046	0.99	0.16	0.0068
	KIR3DL3	−0.045	0.55	0.13	0.66	0.082	0.18
	KIR3DS4	−0.061	0.42	0.37	0.22	0.17	0.0046
Neutrophils	CD66b	0.068	0.36	0.17	0.58	0.041	0.5
	CD11b	−0.01	0.89	0.56	0.046	0.31	2.1e−07
	CCR7	−0.083	0.27	0.16	0.6	0.28	1.8e-6
Th1	T-bet	−0.071	0.34	0.14	0.64	0.21	0.00057
	STAT4	−0.063	0.4	0.52	0.068	0.31	1.7e-07
	TNF-α	0.21	0.0044	0.23	0.45	0.038	0.53
Th2	GATA3	0.032	0.67	0.38	0.2	0.08	0.19
	STAT6	0.11	0.14	0.69	0.0095	0.44	1.5e-14
	STAT5A	−0.009	0.9	0.89	3.8e-05	0.44	1.7e-14
	IL13	0.043	0.56	0.46	0.11	0.08	0.19
Tfh	BCL6	0.31	2.8e-05	0.72	0.0052	0.33	1.8e-08
Th17	STAT3	0.37	7.4e-07	0.87	9.7e-05	0.42	6.2e-13
	IL17A	−0.066	0.38	0.21	0.48	0.1	0.098
T cell exhaustion	PD-1	−0.1	0.16	0.19	0.53	0.22	0.00031
	CTLA4	−0.05	0.5	0.17	0.58	0.22	0.00028
	LAG3	−0.035	0.64	0.4	0.17	0.3	6e-07
	TIM-3	−0.016	0.83	0.59	0.033	0.35	2.1e-09
Mast cells	TPSB2	−0.11	0.14	0.49	0.092	0.12	0.046
	TPSAB1	−0.12	0.1	0.57	0.044	0.17	0.0053
	CPA3	−0.0093	0.9	0.58	0.038	0.32	4.5e-08
	MS4A2	−0.037	0.62	0.34	0.26	0.23	0.00011
	HDC	−0.026	0.72	−0.33	0.28	0.2	0.001

Furthermore, the association between TERF2 expression and chemokines was investigated using the TISIDB database. As depicted in Fig. 4C–K, significant negative correlations were observed between TERF2 expression levels and those of CCL2 (*p* = 0.0082), CCL4 (*p* = 0.035), CCL14 (*p* = 0.0283), CCL15 (*p* = 0.0162), CCL28 (*p* = 0.0207), CXCL2 (*p* = 0.00155), CXCL3 (*p* = 0.0143), CXCL9 (*p* = 0.0374), and CXCL16 (*p* = 0.0109). These findings suggest that TERF2 functions as a negative immunoregulatory factor in ESCC.

### 3.5 TERF2 involved in regulating ESCC cell proliferation through the DDR/P53 signaling pathway

**Fig. 5:**
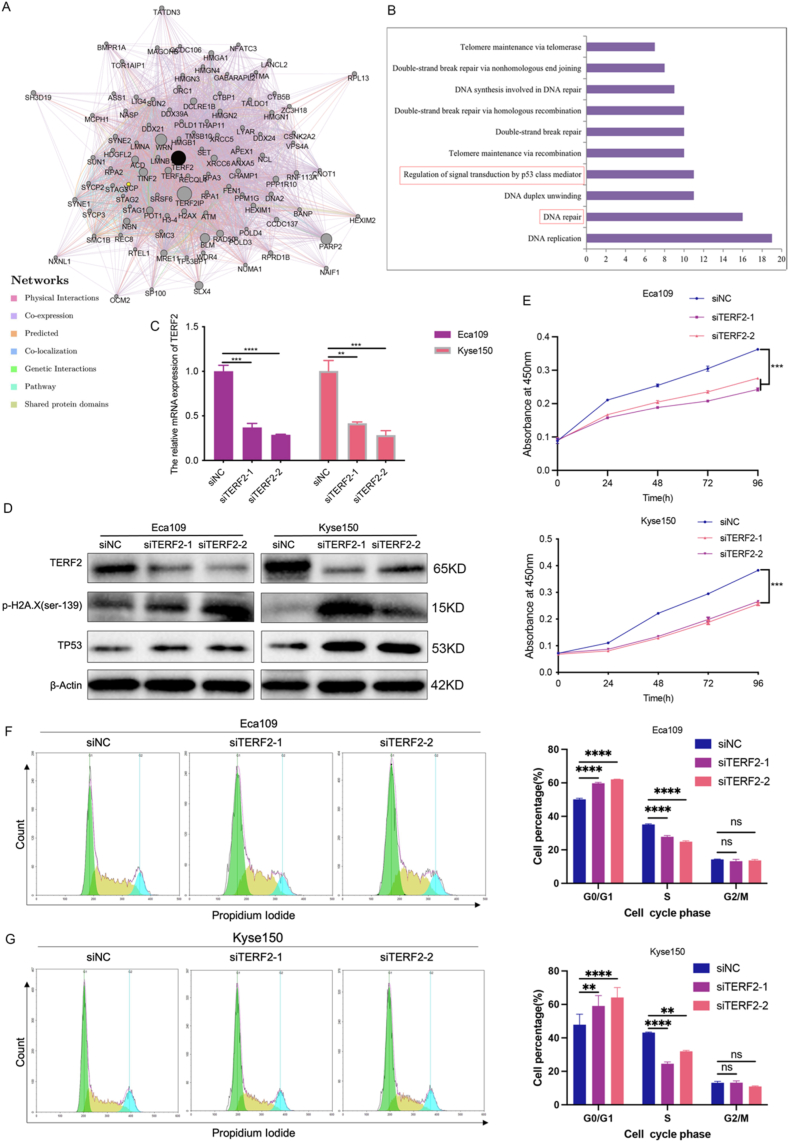
**TERF2 involved in regulating the proliferation of ESCC cells.** A. The network for TERF2, along with the relevant genes examined based on GeneMANIA. B. Biological processes; C. Eca109 and Kyse150 cells transfected with si-NC, siTERF2-1 and siTERF2-2. TERF2 protein and mRNA levels were detected by qRT-PCR assays. D. Downregulation of TERF2 induced the protein expression changes of DDR and TP53 pathways. E. The cell proliferation was determined by CCK8 assay. F-G. Flow cytometry for cell cycle. *p-*value less than 0.01 indicates statistical significance.

A protein-protein interaction (PPI) network centered on TERF2 was constructed using GeneMANIA. Distinctly colored lines within the network represented various interaction types, including co-expression, physical interaction, co-localization, predicted relationships, shared protein domains, pathways, and genetic interactions (Fig. 5A). Subsequent functional enrichment analysis using DAVID revealed a strong association between TERF2 and DNA repair as well as p53 class mediator-regulated signaling (Fig. 5B). To investigate TERF2's functional role, two TERF2 siRNAs were transfected into Eca109 and Kyse150 cells, resulting in a significant reduction of TERF2 expression levels (Fig. 5C–D, Supplementary Fig. 2). To further elucidate the relationship between TERF2 and the DNA damage response (DDR/TP53) signaling pathway, the expression of DNA damage markers H2A.X and TP53 was examined. Notably, protein levels of H2A.X and TP53 were significantly increased following TERF2 knockdown, suggesting that TERF2 depletion induced DNA damage. Furthermore, TERF2 knockdown led to a significant inhibition of ESCC cell viability (Fig. 5E). Cell cycle analysis using flow cytometry revealed a G0/G1 phase arrest and a decreased proportion of cells in the S phase upon TERF2 depletion compared to controls (Fig. 5F–G). These findings suggest TERF2 may regulate ESCC cell proliferation through the DDR/TP53 signaling pathway.

## 4 Discussion

Globally, esophageal squamous cell carcinoma ranks as the eighth most prevalent cancer and the sixth leading cause of cancer-related mortality [21]. Despite continuous advancements in surgical treatment for ESCA, patient survival outcomes remain poor. This is primarily attributed to the paucity of diagnostic markers for early-stage ESCA and the incomplete understanding of the molecular mechanisms driving its initiation and progression. Consequently, there is a pressing imperative need to discover novel diagnostic biomarkers and therapeutic targets for ESCA.

Telomeres, composed of specialized repetitive DNA sequences and associated telomeric proteins at eukaryotic chromosome termini, are essential for safeguarding genomic stability and preventing chromosomal fusion [22]. Structural and functional telomere aberrations have been strongly implicated in aging, hereditary diseases, and cancer development [23]. Notably, frequent aberrant alterations in the expression and genetics of shelterin proteins have been observed in most human malignancies [24]. TERF2 has been found to be overexpressed in colorectal carcinoma [8], breast cancer [25], gastric cancer [6,13], glioblastoma [26], head and neck squamous cell carcinoma [27], hepatocellular carcinoma [10,28,29], acute myeloid leukemia [12], lung cancer [22], renal cell carcinoma [30], skin cancer [31] and oral squamous cell carcinoma [14]. These findings collectively suggest a critical role for TERF2 in tumorigenesis. However, the involvement of TERF2 in ESCC remains relatively unexplored. This study demonstrates significantly elevated TERF2 protein and mRNA levels in ESCA tissues compared to non-cancerous tissues. Furthermore, TERF2 overexpression correlates with patient sex, tumor grade, cancer stage, LNM, and tumor histology. However, larger studies are required to elucidate age-related trends in TERF2 expression. Univariate Cox regression analysis identified TERF2 as an independent prognostic factor for overall survival, with an AUC of 0.744. Furthermore, TERF2 mRNA levels correlate with serum levels of the tumor markers CEA, CYFRA21-1, and TPA. These findings highlight the substantial association between TERF2 and clinical characteristics, suggesting its potential diagnostic utility in ESCA.

Immunological infiltration and the immune microenvironment are critical factors of tumor initiation and progression. Consequently, we investigated the relationships among TERF2 levels, immune infiltration, and cytokine profiles. Advances in immunological research and the elucidation of neoplasm initiation mechanisms have highlighted immune responses and their target molecules as crucial therapeutic targets for ESCA [32]. Chemokines exert a significant influence on the recruitment and localization of immune cells within the TME [33,34]. For instance, Th17 markers (STAT3) are upregulated in cancers [35] and STAT3 signaling often inhibits antitumor immunity and promotes apoptosis resistance [36]. Programmed cell death protein 1 (PD-1), a CD28 family member, is associated with increased tumor invasion and poor survival in ESCC [37]. Overexpression of TRF2 within cancer cells elicits immunosuppressive microenvironment, a phenomenon mediated through the recruitment and accumulation of regulatory T cells [38]. Chemokines and their receptors are well-established drivers of carcinogenic processes, including tumor proliferation, angiogenesis, invasion, and migration [39]. TERF2 downregulates CXCL9 expression in oral carcinoma cell lines [14]. Our study revealed a strong association between TERF2 levels and TIICs (specifically B cells), as well as a broad spectrum of immune markers including CD2, CD3E, CD8A, CD8B, CD19, TNF-α, KIR2DL1, KIR2DL4, KIR3DL1, KIR3DL2, KIR3DS4, CD11b, CCR7, T-bet, STAT3, STAT4, STAT5A, STAT6, PD-1, BCL6, CTLA4, TPSB2, CPA3, LAG3, MS4A2, TIM-3, and HDC, and chemokines such as CCL2, CCL4, CCL14, CCL15, CCL28, CXCL2, CXCL3, CXCL9, and CXCL16. These findings suggest that TERF2 participates in immune responses and the tumor microenvironment in ESCA.

It is well-acknowledged that TERF2 is intricately involved in diverse biological processes and signaling pathways [40]. Emerging evidence indicates that TERF2 interacts with multiple tumor-related pathways. Activation of the Wnt/β-catenin signaling pathway upregulates TERF2 expression, a critical mechanism underlying aberrant cancer cell growth [41]. Furthermore, the RAS/RAF/MEK/ERK signaling cascade regulates TERF2 protein stability within cells [42], and the IκB kinase (IKK) signaling pathway influences TERF2 levels [43]. To elucidate TERF2's potential biological functions, we comprehensively analyzed its role in ESCA. Our study found a strong correlation between TERF2 and the DDR and TP53 signaling pathways. Furthermore, previous studies have demonstrated TERF2's role in modulating proliferation in various tumor types. Inhibition of TERF2 suppresses renal cell carcinoma cell proliferation and migration [30], while TERF2 depletion affects glioblastoma stem cell proliferation [26]. Additionally, TERF2 knockdown significantly reduces oral squamous cell carcinoma xenograft growth in mice, highlighting its potential as a therapeutic target [14]. As a classical tumor suppressor, TP53 regulates cell proliferation through cell cycle control, and its abnormal expression contributes to cancer development [44]. Our findings suggest that TERF2 inhibition induces DNA damage and upregulates TP53 expression. Concurrently, ESCC cell viability is significantly reduced, with an increase in the G0/G1 phase and a decrease in the S phase of the cell cycle. These results suggest TERF2 may inhibit cell proliferation through the DDR/TP53 pathway.

## 5 Conclusion

This study elucidated the expression and clinical significance of TERF2 in ESCA patients, suggesting its potential as a biomarker for ESCA diagnosis and treatment. Our findings indicate that TERF2 may regulate ESCC cell proliferation through the DDR/TP53 signaling pathway. However, the precise biological role and underlying mechanisms of TERF2 in ESCA require further investigation. This constitutes the primary limitation of our study and warrants future research focus.

## Ethics approval and consent to participate

This study gained approval from the Medical Ethics Committee of the Affiliated Hospital of North Sichuan Medical College (File No. 2022ER461-1). Written informed consent was obtained from all participants prior to their involvement.

## Funding

This study was supported by the 10.13039/501100001809National Natural Science Foundation of China (grant no. 82160405), Science and Technology Plan of Jiangxi Province (grant no. 20213BCJ22013， 20212ACB206016).

## Data availability statement

The datasets presented in this study can be found in the online repositories. The names of the repository/repositories and accession numbers (s) can be found in the article materials.

## CRediT authorship contribution statement

**Lihua Yao:** Writing – original draft, Formal analysis, Data curation. **Xinlu Wang:** Data curation. **Zihao Wang:** Software, Methodology. **Xiaozhong Wang:** Writing – review & editing, Funding acquisition. **Xiaolan Guo:** Project administration.

## Declaration of competing interest

The authors declare no conflict of interest, financial or otherwise.

## Figures and Tables

**figs1:**
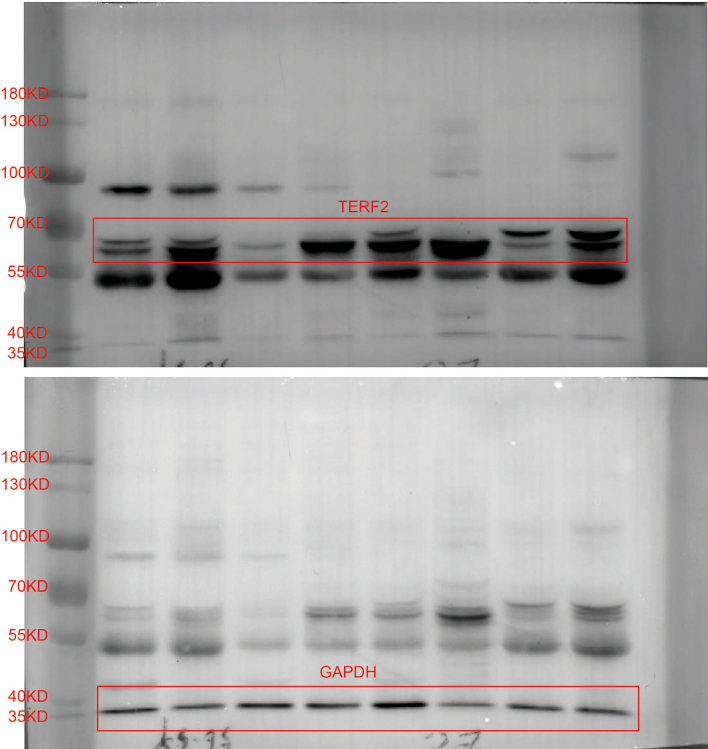


**figs2:**